# Repurposing HLA genotype data of renal transplant patients to prevent severe drug hypersensitivity reactions

**DOI:** 10.3389/fgene.2023.1289015

**Published:** 2023-10-16

**Authors:** Lisanne E. N. Manson, Sander J. Delwig, Jos J. M. Drabbels, Daan J. Touw, Aiko P. J. De Vries, Dave L. Roelen, Henk-Jan Guchelaar

**Affiliations:** ^1^ Department of Clinical Pharmacy and Toxicology, Leiden University Medical Center, Leiden, Netherlands; ^2^ Department of Immunohematology, Leiden University Medical Center, Leiden, Netherlands; ^3^ Department of Clinical Pharmacy and Pharmacology, University Medical Center Groningen, University of Groningen, Groningen, Netherlands; ^4^ Division of Nephrology, Department of Internal Medicine, Leiden University Medical Center, Leiden, Netherlands; ^5^ Leiden Transplant Center, Leiden University Medical Center, Leiden, Netherlands

**Keywords:** pharmacogenetics, HLA, hypersensitivity, NGS, SCARs, kidney transplant patients

## Abstract

**Introduction:** Specific alleles in human leukocyte antigens (HLAs) are associated with an increased risk of developing drug hypersensitivity reactions induced by abacavir, allopurinol, carbamazepine, oxcarbazepine, phenytoin, lamotrigine, or flucloxacillin. Transplant patients are genotyped for HLA as a routine practice to match a potential donor to a recipient. This study aims to investigate the feasibility and potential impact of repurposing these HLA genotype data from kidney transplant patients to prevent drug hypersensitivity reactions.

**Methods:** A cohort of 1347 kidney transplant recipients has been genotyped in the Leiden University Medical Center (LUMC) using next-generation sequencing (NGS). The risk alleles HLA-A*31:01, HLA-B*15:02, HLA-B*15:11, HLA-B*57:01, and HLA-B*58:01 were retrieved from the NGS data. Medical history, medication use, and allergic reactions were obtained from the patient’s medical records. Carrier frequencies found were compared to a LUMC blood donor population.

**Results:** A total of 13.1% of transplant cohort patients carried at least one of the five *HLA* risk alleles and therefore had an increased risk of drug-induced hypersensitivity for specific drugs. HLA-A*31:01, HLA-B*15:02, HLA-B*57:01, and HLA-B*58:01 were found in carrier frequencies of 4.61%, 1.19%, 4.46%, and 3.35% respectively. No HLA-B*15:11 carrier was found. In total nine HLA-B*57:01 carriers received flucloxacillin and seven HLA-B*58:01 carriers within our cohort received allopurinol.

**Discussion:** Our study shows that repurposing HLA genotype data from transplantation patients for the assignment of HLA risk alleles associated with drug hypersensitivity is feasible. The use of these data by physicians while prescribing drugs or by the pharmacist when dispensing drugs holds the potential to prevent drug hypersensitivity reactions. The utility of this method was highlighted by 13.1% of the transplant cohort patients carrying an actionable HLA allele.

## 1 Introduction

An adverse drug reaction (ADR) is defined by the World Health Organization as “a response to a drug which is noxious and unintended, and which occurs at doses normally used in humans for the prophylaxis, diagnosis, or therapy of disease, or for modifications of physiological function” (World Health Organisation, 1972). ADRs represent a major burden in healthcare, 3.5% of all hospital admissions were found to be caused by ADRs and 10.1% of patients experienced an ADR during hospitalization (Bouvy et al., 2015).

Pharmacogenetics has the potential to reduce ADRs by identifying patients that are at risk. Efforts in pharmacogenetic screening have proven its potential. In a recent multicenter, study in 6944 patients, the PREPARE study from the Ubiquitous Pharmacogenomics (U-PGx) consortium, preventive screening of a 12-gene panel reduced the risk of clinically relevant adverse drug reactions by 30% (Swen et al., 2023). In the PREPARE study of the HLA risk alleles only the HLA-B*57:01 (HLA) risk allele was included.

Human Leukocyte Antigens (HLAs) are important in immunology and clinical medicine. The function of HLA-encoded proteins is to distinguish endogenous from exogenous particles. HLA molecules also play a crucial role in transplant organ acceptance. HLA mismatching has been shown to be a good predictor for allograft rejection, which is why genotyping and serotyping has become the standard of care before a transplantation procedure. In addition, several HLA alleles have been identified as “risk alleles” that pose an increased chance of hypersensitivity with certain drug-gene combinations. Hypersensitivity reactions occur when an aberrant immune response is generated, this can lead to reactions with clinical manifestations that range from mild to very severe.

Over the last ten to 15 years, the contribution of pharmacogenetic profiles to the prevalence of ADRs has been gradually elucidated and guidelines have been established (Swen et al., 2008; Swen et al., 2011). The Dutch Pharmacogenetics Working Group (DPWG) aims to provide guidelines for therapeutic recommendations based on pharmacogenetic research. These guidelines include pharmacokinetic and pharmacodynamic drug-gene interactions to prevent Type A adverse drug reactions but also includes type B idiosyncratic drug reactions that have been associated with specific HLA alleles. There are five known HLA risk alleles in the Dutch guidelines:” HLA-A*31:01, HLA-B*15:02, HLA-B*15:11, HLA-B*57:01, and HLA-B*58:01 (Farmacogenetica and KNMP Kennisbank, 2023).

Stevens-Johnson syndrome and toxic epidermal necrolysis (SJS/TEN) are severe cutaneous adverse reactions that are rare and potentially life-threatening. Incidences of SJS/TEN can range from two to seven cases per million people per year (Charlton et al., 2020). Both hypersensitivity reactions are characterized by detachment of the epidermis and mucous membrane with SJS being <10% detachment, SJS-TEN 10%–30%, and TEN >30% (Frantz et al., 2021). Reports for mortality rates for SJS and TEN differ, with a mortality rate of 4.8%–9% for SJS, 19.4%–29% for SJS/TEN, and 14.8%–48% for TEN. HLA-B*15:02 or HLA-B*15:11 carriers initiating carbamazepine, lamotrigine, phenytoin, or oxcarbazepine are at higher risk of developing SJS/TEN, as well as HLA-B*58:01 carriers initiating allopurinol (Hung et al., 2006; Yip et al., 2012; Cheung et al., 2013; Wu et al., 2016; Asgarpour et al., 2021).

Another ADR that is also associated with HLA-B*15:02 and anti-epileptic drugs and HLA-B*58:01 and allopurinol, but also in carbamazepine initiators with HLA-A*31:01 is drug reaction with eosinophilia and systemic symptoms (DRESS) (Yip et al., 2012; Wu et al., 2016). DRESS typically occurs within 2 weeks to 2 months after the first administration of a drug. The hypersensitivity reaction is diagnosed by an acute rash, suspected drug-related reaction, fever, atypical lymphocytosis and/or eosinophilia, involvement of >1 organ, and lymphadenopathy at more than 2 sites (Kardaun et al., 2007). Due to the variety in clinical presentation, the true incidence is unknown, but DRESS has been reported at around 10 per million. DRESS is more commonly found in females than in males in a ratio of 1.25:1. A mortality rate of 2% was found for DRESS (Kardaun et al., 2013). But after treatment of DRESS patients are at risk of relapse and sequelae for weeks and sometimes months, which adds to the severity of the hypersensitivity reaction.

HLA-B*57:01 carriers starting on flucloxacilline have an increased risk of developing drug induced liver injury (DILI) (Daly et al., 2009). DILI accounts for up to 15% of cases of acute liver failure (Aithal et al., 2011). DILI is diagnosed by measuring at least one of the following: more than or equal to fivefold elevation above the upper limit of normal (ULN) alanine aminotransferase (ALT), more than or equal to twofold elevation above ULN for alkaline phosphatase or more than equal to threefold elevation in ALT concentration and simultaneous elevation of bilirubin concentration exceeding 2x ULN. HLA-B*57:01 is also associated with abacavir hypersensitivity syndrome, and HLA testing is mandatory before initiation of abacavir since the drug is contraindicated in HLA-B*57:01 carriers (Mallal et al., 2008).

The unpredictability and severity of hypersensitivity reactions related to HLA highlight the need for preventive measures. HLA screening has been the standard of care for matching donors and recipients for transplantations for a considerable time. This available HLA genotype data from the transplantation matching mitigates the cost constraints of selective preventive screening for HLA hypersensitivity reactions. Other studies have shown that diagnostic data can be repurposed to give clinical guidance in the context of preventing drug-gene interactions (van der Lee et al., 2020). This study aims to evaluate the feasibility of repurposing the readily available HLA data from transplant patients. Furthermore, the impact of including this data in the patient’s medical file for preventing HLA-associated drug hypersensitivity reactions will be investigated.

## 2 Materials and methods

### 2.1 Patient population

This study is a single-center retrospective study. The cohort consists of renal transplant patients from the Leiden University Medical Center (LUMC) transplant center that were genotyped before kidney transplantation between 2019 and the 23rd of December 2022. Genotyping data was collected from the LUMC transplantation center database. The protocol of this study was approved by the Medical Research Ethics Committee (nWMO Commissie Divisie 4) of the Leiden University Medical Center under reference number nWMO-D4-2022-026. They concluded that Medical Research Involving Human Subjects Act (Dutch abbreviation: WMO) does not apply to this study and therefore it was exempt from full review by the Institutional Review Board.”

### 2.2 Genotyping data

The variants that were selected for repurposing were the 5 HLA variants included in the DPWG and/or Clinical Pharmacogenetics Implementation Consortium (CPIC) guidelines. These HLA variants in the guidelines are supported by multiple studies and have been incorporated into the Dutch clinical decision support systems. Patients of the LUMC transplantation center are sequenced using amplicon sequencing. The sequencing method used by the LUMC transplant center is the Illumina NGS method. The HLA amplicons were generated using the GenDx NGSgo-MX11-3 kit (GenDX, 2023). The sequencing instrument used was the MiSeq from Illumina. The MiSeq uses the sequencing by synthesis (SBS) technique which tracks the addition of labeled nucleotides as the DNA is copied. This method allowed for sequencing both the coding and non-coding regions. After sequencing, Fastq files were analyzed by specific HLA software NGS-Engine from the company GenDx Utrecht the Netherlands. Data was obtained from the patient’s medical record. For patients carrying a risk allele, age, and sex were collected. The number of heterozygous and homozygous risk allele carriers was extracted from the data. Homozygous risk allele carriers, heterozygote risk allele carriers, and non-carriers were tested for deviations from Hardy-Weinberg equilibrium in Rstudio version 4.2.1. with Hardy-Weinberg package 1.7.5 according to an article by Graffelman (Graffelman, 2015). Differences between the expected carriers and observed carriers were statistically tested using a χ^2^ goodness-of-fit test in Rstudio version 4.2.1.

### 2.3 Medication data

The medication use of HLA risk allele carriers was manually reviewed in the patient’s electronic medical record. Access to “Landelijk Schakelpunt” or National Exchange Point data was requested and granted, this allowed access to all previously prescribed medication available on the 23rd of December 2022. Electronic medical records were searched for the use of drugs for which there is a higher chance of drug hypersensitivity.

### 2.4 Hypersensitivity reactions

Patients who carried an HLA risk allele and were dispensed the corresponding risk drug were exploratively evaluated for HLA-associated hypersensitivity reactions. Recorded allergies were searched for in the electronic medical records, and patients’ notes were manually reviewed with search terms such as “allergic,” “rash,” and “fever.” The laboratory results for patients with HLA-B*57:01 and flucloxacillin were checked for DILI by searching for ALT, ALP, and bilirubin serum concentrations.

### 2.5 Population comparison

To test whether the LUMC transplantation center cohort (*n* = 1345) was representative of the Dutch population, the carrier frequencies of the risk alleles were compared to a LUMC blood donor population (*n* = 1305) and the average of three Dutch populations. Allele frequencies of the LUMC blood donor population and a “Deutsche Knochenmarkspenderdatei” (DKMS) minority were retrieved from The Allele Frequency Net Database (Gonzalez-Galarza et al., 2020). In addition, after a PubMed search for populations that had been screened for HLA a Dutch population was found on PubMed and was included in the average of the Dutch populations (Hou et al., 2019). HLA carrier frequencies of the risk alleles were compared using a Chi-squared statistical test in Rstudio version 2022.07.1 + 554.

## 3 Results

### 3.1 Study population

The study population consisted of a total of 1347 renal transplant patients recruited from the LUMC transplant center. One patient was excluded from the study because the patient potentially carried HLA-A*31:01 and HLA-B*57:01, but genotyping results were inconclusive. One patient was excluded because no genotyping data was available. The average age of the patient group was 57.9 years old. The median age of the patient group was 60 years old, and the interquartile range (IQR) was 49–69. In the original study group, 59% of patients were males and 41% of patients were females**.**

### 3.2 Allele frequencies

A total of 188 risk alleles were found in the study population (Table 1). 13.1% of patients (176/1345) carried at least one actionable HLA gene. HLA-A*31:01, HLA-B*57:01, and HLA-B*58:01 were found most often in the patient population. They had an allele frequency of respectively 2.3%, 2.2%, and 1.8% of all alleles. HLA-B*15:02 was found with an allele frequency of 0.7% of all alleles. HLA-B*15:11 was not found in the study population. 12 patients carried multiple risk alleles of which five carried both HLA-A*31:01 and HLA-B*57:01, two carried both HLA-A*31:01 and HLA-B*58:01, one carried HLA-B*15:02 and HLA-B*58:01, one carried HLA-B*57:01 and HLA-B*58:01 and three patients where homozygous for HLA-B*58:01.

**TABLE 1:** HLA risk allele frequencies and carrier frequencies in the LUMC transplant center population (*n* = 1345).

Allele	Allele count	Allele frequency	HLA carrier frequency
HLA-A*31:01	62	0.0230	0.0461
HLA-B*15:02	18	0.0067	0.0134
HLA-B*15:11	0	0.0000	0
HLA-B*57:01	60	0.0223	0.0446
HLA-B*58:01	48	0.0178	0.0335
Total	188	0.0698	0.1389

### 3.3 Medical history

Twenty-three patients were excluded from the drug hypersensitivity study due to the unavailability of pharmacy file data. A total of 17 patients were found that carried an HLA risk allele and used its corresponding risk drug (Table 2). 10 patients who had HLA-B*57:01 received prior flucloxacillin treatment and 7 patients who had HLA-B*58:01 received allopurinol treatment (Table 2). None of the patients that had a risk allele and received a risk drug developed one of the drug hypersensitivity reactions described in the DPWG guidelines.

**TABLE 2:** HLA risk allele carriers from the LUMC transplant center who also used the corresponding risk medication. None of the patients who carried a risk allele suffered the corresponding drug hypersensitivity reaction.

Allele	Risk medication	Number of patients that received risk drugs out of total carriers	Drug hypersensitivity reactions
HLA-A*31:01	Carbamazepine	0/57	SJS/TEN, DRESS, MPE
HLA-B*15:02	Carbamazepine, oxcarbazepine, lamotrigine, phenytoin	0/16	SJS/TEN
HLA-B*15:11	Carbamazepine	0/0	SJS/TEN
HLA-B*57:01	Abacavir	0/47	ABC-HSS
HLA-B*57:01	Flucloxacillin	10/47	DILI
HLA-B*58:01	Allopurinol	7/42	SJS/TEN, DRESS

### 3.4 Hardy-Weinberg equilibrium

Heterozygous risk allele carriers, homozygous risk allele carriers, and non-carriers were tested for deviations from Hardy-Weinberg equilibrium. Statistical difference was found for HLA-B*58:01 (*p* < 0.001) and no statistical difference was found for the other alleles (*p*-value HLA-A*31:01: 0.387, HLA-B*15:02: 0.805 and HLA-B*57:01: 0.403).

### 3.5 Population comparison

The carrier frequency of the risk alleles was compared between the populations.

A statistical difference in carrier frequency between our population and the LUMC blood donor population was found for all HLA risk alleles. The most significant difference was found in the risk alleles *HLA-B:15:02* and HLA-B*58:01 (Table 3). A significant difference was also found for HLA-A*31:01 and HLA-B*57:01. For the comparison with the average of three Dutch populations the results were similar (Table 3).

**TABLE 3:** LUMC transplantation center, LUMC blood donor population calculated carriers, and average HLA risk allele carriers in 3 Dutch populations. *p*-values for the comparison of the LUMC transplantation center.

	Transplantation center population (*n* = 1345)	Blood donor population (*n* = 1305)		Three Dutch populations’ average	
	Risk allele carriers	Risk allele carriers	*p*-value	Risk allele carriers	*p*-value
HLA-A*31:01	4.60%	6.51%	0.040	6.67%	0.028
HLA-B*15:02	1.33%	0	<0.001	0.08%	0.002
HLA-B*15:11	0	0	-	0	-
HLA-B*57:01	4.46%	6.67%	0.002	6.60%	0.022
HLA-B*58:01	3.35%	1.23%	<0.001	1.38%	<0.001

## 4 Discussion

In our study group we found that 13.1% of all patients carried at least one risk allele. HLA-A*31:01, HLA-B*57:01 and HLA-B*58:01 were found the most. HLA-B*15:02 was also found but in a lower percentage. No HLA-B*15:11 carriers were found. 17 patients in our study population carried a risk allele and received the corresponding risk medication. 10 patients received flucloxacillin and carried the HLA-B*57:01 risk allele. 7 patients received allopurinol and carried the HLA-B*58:01 risk allele. No patients were found that developed drug hypersensitivity reactions. All risk alleles found will be recorded in the patient’s electronic health records after which actionable pharmacogenomics alerts will be generated during prescribing and dispensing drugs. In The Netherlands, automated medication surveillance is in operation in hospitals, by general practitioners and pharmacists. Most of the automated medication systems use the G-standard, a national electronic drug database. The DPWG guidelines are incorporated in the G-Standard as of 2006. By recording the five *HLA* risk alleles as a contra-indication in the electronic health record, actionable alerts will be generated. In this study we focused on the five *HLA* risk alleles incorporated in the DPWG guidelines. The results show that re-using renal transplant *HLA* genotype data is feasible. Despite no drug hypersensitivity reactions being found, a relatively large percentage of patients carried at least one risk allele and could thus receive clinically relevant advice possibly preventing future hypersensitivity reactions.

Repurposing genetic data for use in pharmacogenetics may be an interesting approach to stimulate the implementation of personalized medicine. Indeed, in a previous study, we showed the feasibility of repurposing pharmacogenetic variants of the U-PGx Passport (van der Wouden et al., 2019) from existing whole exome sequencing data (van der Lee et al., 2020). Another recently published study also repurposed HLA-A*31:01 and HLA-B*15:02 genotype data from whole genome sequencing. This study selected patients with epilepsy from the Genomics England UK 100 000 Genomes Project. Our study looked more broadly at this by repurposing five *HLA* variants in a transplant center cohort.

Since impaired renal function is a known risk factor for drug hypersensitivity reactions (Park et al., 2019), reusing the HLA risk allele data may be especially useful for kidney transplant patients. The risk of developing allopurinol-induced severe cutaneous adverse drug reactions is 1.6%–2.0% for HLA-B*58:01 carriers*,* for patients with chronic renal insufficiency the risk is increased to 8%–18% to develop a SCAR (Jung et al., 2014; Park et al., 2019). This means that this group of patients is inherently more susceptible than the average population to develop drug hypersensitivity reactions and thus HLA risk allele data on this group is more valuable.

All patients selected for the study were kidney transplant patients transplanted in the LUMC transplant center. The distribution of homozygotes and heterozygotes for risk alleles was tested following Hardy-Weinberg equilibrium. From the results, HLA-B*58:01 carriers were the only group that deviated significantly from the expected carriers. This deviation may have been caused by the three homozygous HLA-B*58:01 carriers. An explanation for the deviation from Hardy-Weinberg equilibrium may be population admixture. Indeed, in the Netherlands, nearly 12 percent of inhabitants had one foreign-born parent (CBS, 2023) and it is therefore likely that the transplant center group had a different range of ethnicities than the comparative. Unfortunately, we are unable to confirm this hypothesis as no ethnicity data from our study group was available.

The positive predictive value (PPV) for developing HLA-B*57:01-associated flucloxacillin-induced DILI is 0.11% (Daly et al., 2009), and for HLA-B*58:01-associated allopurinol-induced SJS/TEN the PPV is 5.5% (Lonjou et al., 2008). So, to find one flucloxacillin-induced DILI patient we would theoretically need 909 HLA-B*57:01 positive patients. Therefore, it is not surprising that we did not find a patient with flucloxacillin-induced hypersensitivity. For allopurinol, an estimated 18 HLA-B*58:01 positive patients would be required to find one case of SJS/TEN. Considering the risk allele carrier frequency in The Netherlands, and the low positive predictive value, the study population was relatively small for finding one of the HLA-associated drug hypersensitivity reactions. To increase the chance of finding a drug hypersensitivity reaction, the sample size should be increased by collecting data over a longer period. An increase in the sample size would also increase the chance of finding an HLA-associated drug hypersensitivity reaction and thus increase the power of the study. Nevertheless, since the pharmacogenetic data are repurposed the fact that the PPV is relatively low is of less importance.

When repurposing NGS data, ideally you would want to include all known risk HLA alleles. For instance, there are many other class I HLA risk alleles but also examples of class II HLA risk alleles such as HLA-DRB1*11:01 and statin induced autoimmune myopathy or HLA-DQB1*06:02 and amoxicillin induced DILI. In this study we focused only on those alleles already present in DPWG pharmacogenetics guidelines and for which the infrastructure for pharmacogenetics alerts is already present in The Netherlands (Lucena et al., 2011; Mammen et al., 2012).

The identification of drug hypersensitivity reactions was difficult since the drug hypersensitivity reactions were not registered and collected systematically but extracted from the patient’s electronic medical record notes. However, because of the severity of DRESS, ABC-HSS, and SJS/TEN, it was expected that these reactions could be found in the electronic medical records. DILI symptoms can be objectified by measuring liver enzyme levels such as ALT, ALP, or bilirubin. After the first year kidney transplant patients are transferred to aftercare, where they are routinely tested for liver enzymes. However, this data is not available in the LUMC, which made it harder for us to find cases of DILI. More generally, the diagnosis of all drug hypersensitivity reactions is difficult because of the late onset of symptoms.

Repurposing available HLA data from the transplant center was found to be an efficient way to collect pharmacogenetic data. Repurposing can be an attractive approach, especially in the case of variants with suboptimal test criteria (sensitivity, specificity) such as HLA variants (Manson et al., 2020) but potentially severe clinical outcomes. Indeed, information on HLA risk alleles is very valuable because the drug hypersensitivity reactions associated with HLA have a very high severity. The mortality of the drug hypersensitivity reactions ranges from 0.07% to 34%. ABC-HSR has the lowest mortality of 0.07% (Mounzer et al., 2019) and SJS at 24% (Sekula et al., 2013), and SJS/TEN at 34% (Sekula et al., 2013) has the highest mortality rate. DILI and DRESS have a mortality rate of 7.6% (Hayashi et al., 2017) and 2% (Kardaun et al., 2013) respectively. Mortality is not the only severe clinical outcome that can result from these drug hypersensitivity reactions. Patients can develop long-term complications after SJS/TEN. These include cutaneous, mucosal, ocular, visceral, and psychologic complications (Lee et al., 2013). These side effects can also, in varying degrees, reduce the quality of life for patients after they have been discharged from the hospital.

In conclusion, our study shows that repurposing HLA genotype data from transplant recipients for the assignment of HLA risk alleles associated with drug hypersensitivity is feasible. Our study showed that a significant percentage of patients carried at least one actionable genotype. In this group, relatively high carrier frequencies were found for HLA-B*15:11 and HLA-B*58:01 and relatively low carrier frequencies for HLA-A*31:01 and HLA-B*57:01 making this group unique. In our cohort several patients were found that could have potentially developed a drug hypersensitivity reaction after having both the HLA risk allele and having received the corresponding risk drug. Although the prevalence of these drug hypersensitivity reactions is low, their severity warrants documentation of these risk alleles from readily available data sources in the electronic health record. Because the use of this data by physicians while prescribing drugs, or by the pharmacist when dispensing drugs holds the potential to prevent severe drug hypersensitivity reactions.

## Data Availability

The data analyzed in this study is subject to the following licenses/restrictions: Clinical data from the Electronical Medical Records of patients was used for this study. Requests to access these datasets should be directed to h.j.guchelaar@lumc.nl.
